# *BrCNGC12* and *BrCNGC16* mediate Ca^2+^ absorption and transport to enhance resistance to tipburn in Chinese cabbage

**DOI:** 10.1111/pbi.70113

**Published:** 2025-05-03

**Authors:** Jingping Yuan, Changwei Shen, Ruixiang Chen, Yunduan Qin, Shuai Li, Bo Sun, Chunyang Feng, Xinlei Guo

**Affiliations:** ^1^ School of Horticulture and Landscape Architecture Henan Institute of Science and Technology Xinxiang 453003 China; ^2^ Henan Engineering Research Center of the Development and Utilization of Characteristic Horticultural Plants Xinxiang 453003 China; ^3^ School of Resources and Environmental Sciences Henan Institute of Science and Technology Xinxiang 453003 China

**Keywords:** Chinese cabbage, tipburn, CNGCs, virus‐induced gene silencing, overexpression

## Abstract

Tipburn is a common physiological disorder in leafy vegetables, significantly impairing crop growth and commercial value. It is widely recognized that Ca^2+^ deficiency is a key factor triggering tipburn; however, the functions and regulatory mechanisms of genes conferring resistance remain largely unexplored. Through transcriptomic analysis of Chinese cabbage under normal (medium calcium, MCa) and Ca^2+^‐deficient (low calcium, LCa) conditions, we observed that genes in the hormone and calcium signalling pathways exhibited significant responses to LCa stress. Among these, the cyclic nucleotide‐gated ion channel (*CNGC*) genes *BrCNGC12* and *BrCNGC16*, part of the calcium signalling pathway, were notably up‐regulated and down‐regulated, respectively, under LCa stress. Silencing BrCNGC12 in Chinese cabbage improves Ca^2+^ absorption and distribution, which strengthens tipburn resistance. Conversely, under LCa stress, heterologous expression of *BrCNGC16* in *Arabidopsis thaliana* increases resistance to tipburn, whereas partial silencing of *BrCNGC16* in Chinese cabbage diminishes resistance, with both outcomes linked to altered Ca^2+^ uptake and translocation. Additionally, overexpression of *BrCNGC16* in Chinese cabbage promotes Ca^2+^ uptake and translocation, thereby enhancing resistance to tipburn and mitigating oxidative damage induced by Ca^2+^ deficiency. In conclusion, *BrCNGC12* and *BrCNGC16* play pivotal roles in tipburn resistance in Chinese cabbage, offering novel insights into the interplay between the calcium signalling pathway and tipburn resistance.

## Introduction

Chinese cabbage, a member of the *Brassica rapa* genus within the Brassicaceae family with a long history of cultivation, is a globally significant economic vegetable. It occupies an estimated 2.67 million hectares of planting area annually in China, accounting for 15% of the national vegetable planting area and contributing nearly 60 billion yuan in economic value (Yuan *et al*., 2021). Tipburn in Chinese cabbage, a prevalent physiological disorder in production, has an average incidence rate of 10%–30%, potentially exceeding 80% in severe cases, causing substantial economic losses for growers and showing an increasing trend in severity, as reported by Su *et al*. (2019). Recognized as a physiological disorder, tipburn in Chinese cabbage is primarily driven by Ca^2+^ deficiency in plants, attributable to both direct and indirect factors (Kuo *et al*., 1981). Indirect factors involve insufficient Ca^2+^ availability in the external environment, which impairs plant growth, while direct factors stem from the plant's limited ability to absorb Ca^2+^ adequately, resulting in stunted growth due to internal Ca^2+^ insufficiency (Saure, 1998). This Ca^2+^‐related physiological disorder occurs frequently and has significantly threatened the yield and quality of Chinese cabbage in recent years (Su *et al*., 2019).

Ca^2+^ is an essential nutrient for plant growth and development, playing pivotal roles in seed germination, flowering, fruiting, photosynthetic phosphorylation, electron transport, cell motility and hormone regulation (Edel *et al*., 2017). Moreover, Ca^2+^ is a critical component in stabilizing cell membranes and cell walls, contributing to enzyme regulation and osmotic adjustment (Lötze *et al*., 2008). As a key second messenger in the cytoplasm, Ca^2+^ also plays a vital role in plant stress resistance mechanisms (Edel *et al*., 2017). Ca^2+^ deficiency commonly leads to physiological disorders in fruits and vegetables, causing wilting and necrosis of tender tissues, such as young leaves and shoot tips (Al Shoffe *et al*., 2019). This occurs due to two primary reasons: first, Ca^2+^ is preferentially transported to actively growing organs, such as leaves, where it becomes relatively immobile and challenges redistribution to inner leaves, leading to Ca^2+^ deficiency in those tissues (Lötze *et al*., 2008). Second, insufficient Ca^2+^ in cell membranes and walls compromises selective permeability, resulting in leakage of intracellular solutes and subsequent cell damage or death (Lötze *et al*., 2008).

In recent years, progress has been made in understanding how plants absorb and transport Ca^2+^. Studies have demonstrated that the interplay among Ca^2+^ channels, exchangers and pumps plays a pivotal role in the uptake and translocation of Ca^2+^ (Tian *et al*., 2019). Cyclic nucleotide‐gated channels (CNGCs) are key regulators of Ca^2+^ influx (Wang *et al*., 2019). Transcriptomic analysis has shown that the expression levels of seven *CNGCs* in pear tree leaves were significantly up‐regulated following 2 days of Ca^2+^‐deficiency treatment (Sun *et al*., 2021). Changes in Ca^2+^ concentration are tightly linked to alterations in Ca^2+^ sensors, such as calmodulins (CaMs), calcineurin B‐like proteins (CBLs) and calcium‐dependent protein kinases (CDPKs/CPKs) (Dodd *et al*., 2010; Zhang *et al*., 2022). Moreover, research has revealed that hormones including auxin (IAA), salicylic acid (SA), jasmonic acid (JA), abscisic acid (ABA) and brassinosteroids are implicated in tipburn (Chen *et al*., 2016; Li *et al*., 2022, 2023). Certain transcription factor families (TFs), such as MYB, WRKY, bZIP, MADS‐box, GT (L), NAC, AGL, SCL and NAM, have been indirectly shown to respond to low‐calcium (LCa) stress (Michailidis *et al*., 2020). These studies collectively suggest that these genes may play significant roles in the uptake and translocation of Ca^2+^.

In Chinese cabbage, several reports have identified genes involved in defending against Ca^2+^‐deficiency‐induced tipburn. For example, studies have indicated that Ca^2+^ transport‐related genes, such as *ACA* (*P2B‐type Ca* ^*2*+^ *‐ATPase pump*), *CAX* (*Ca* ^*2*+^/*H* ^+^ *antiporter*) and *ECA* (*P2A‐type Ca* ^*2*+^ *‐ATPase pump*), contribute to the defence mechanisms against Ca^2+^‐deficiency‐induced tipburn (Cui *et al*., 2023; Lee *et al*., 2016). Furthermore, circRNA‐seq analysis has suggested that specific circRNAs may be involved in the response of Chinese cabbage to Ca^2+^‐deficiency‐induced tipburn (Wang *et al*., 2019). Through sequence variation comparison, molecular marker verification and transgenic studies, researchers have identified the calcium‐wire protein BrCRT2 (Calreticulin 2) as a central gene in the interplay between the Ca^2+^ pathway and tipburn resistance in Chinese cabbage (Su *et al*., 2019). Regarding CNGCs, investigations using extreme tipburn‐resistant and tipburn‐sensitive Chinese cabbage lines, coupled with resequencing analysis, have uncovered a strong association between tipburn and mutations in CNGCs (Liu *et al*., 2017). However, detailed functional studies on the role of *CNGCs* in Ca^2+^ uptake and translocation in Chinese cabbage remain unreported.

In this study, the tipburn‐sensitive Chinese cabbage variety ‘HK‐8’ was selected as the experimental material to examine transcriptomic data from the inner leaves of seedlings under medium‐calcium (control, MCa) and low‐calcium (LCa) conditions. Based on Gene Ontology (GO) and Kyoto Encyclopedia of Genes and Genomes (KEGG) pathway analyses, Ca^2+^‐related differentially expressed genes (DEGs) were identified, and functional validation was performed on the key candidate genes *BrCNGC12* and *BrCNGC16*. These findings will provide novel insights into the relationship between *CNGCs* and tipburn resistance.

## Results

### LCa‐induced tipburn disease in Chinese cabbage

**Figure 1:**
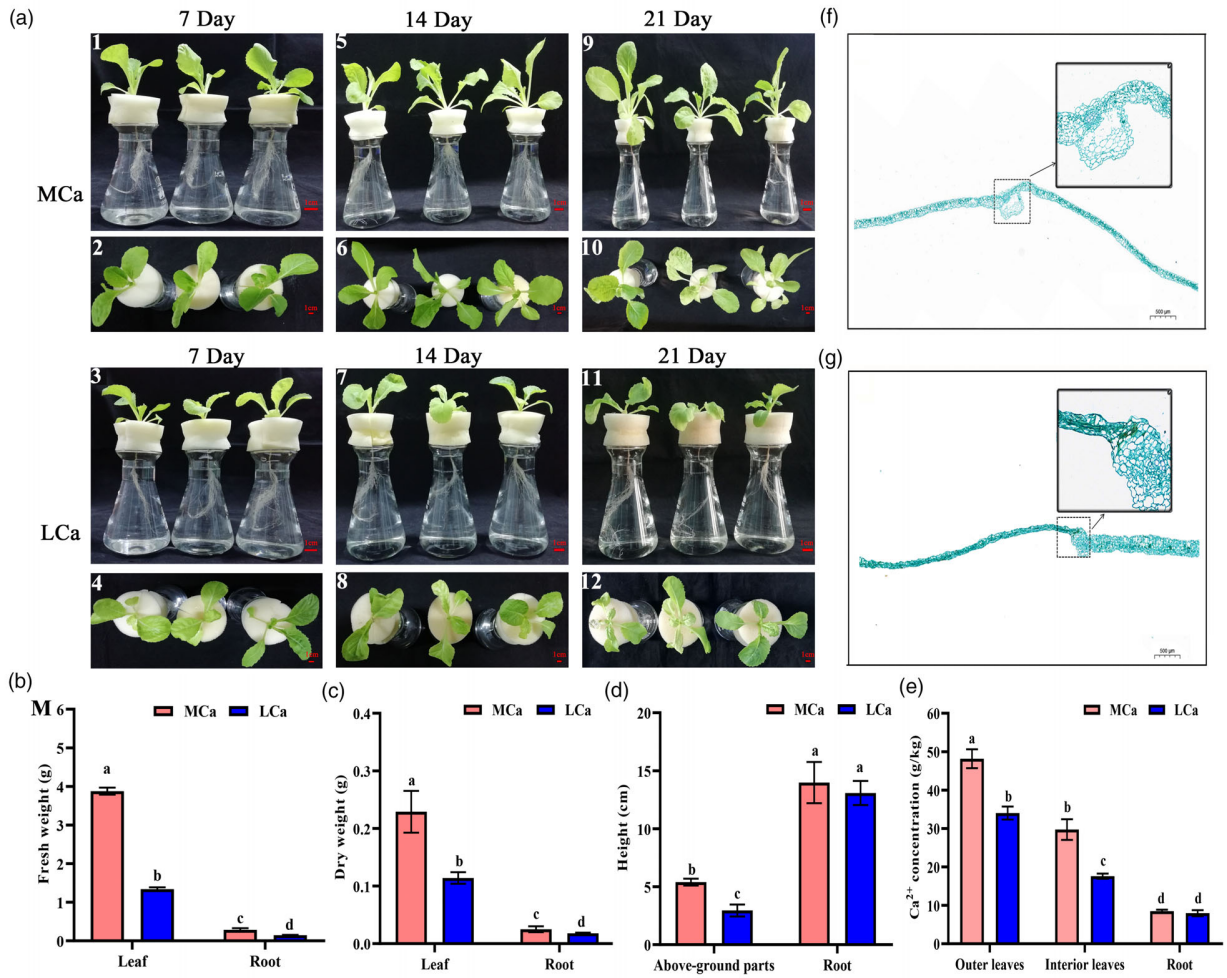
The effects of MCa and LCa treatments on Chinese cabbage seedlings. (a) Phenotypes of Chinese cabbage seedlings under MCa and LCa treatments; 1–2, phenotype of Chinese cabbage at 7 days after MCa treatment (lateral and top views); 3–4, phenotype of Chinese cabbage at 7 days after LCa treatment (lateral and top views); 5–6, phenotype of Chinese cabbage at 14 days after MCa treatment (lateral and top views); 7–8, phenotype of Chinese cabbage at 14 days after LCa treatment (lateral and top views); 9–10, phenotype of Chinese cabbage at 21 days after MCa treatment (lateral and top views); 11–12, phenotype of Chinese cabbage at 21 days after LCa treatment (lateral and top views). Each treatment was repeated five times, and 3 plants were selected for each repetition. The bar represents 1 cm. (b) Fresh weight of the inner leaves and root systems of Chinese cabbage seedlings under MCa and LCa treatments. (c) Dry weight of the inner leaves and root systems of Chinese cabbage seedlings under MCa and LCa treatments. (d) Root length and plant height of Chinese cabbage seedlings under MCa and LCa treatments. (e) Ca^2+^ concentrations in the outer leaves, inner leaves and roots of Chinese cabbage under MCa and LCa treatments; data in (b–e) represent the mean of 5 biological replicates and 3 technical replicates, with error bars indicating the standard deviation (SD) of the mean. Different letters above the bars indicate significant differences (*P* < 0.05). (f) Anatomical structure of the inner leaves of Chinese cabbage under MCa stress. (g) Anatomical structure of the inner leaves of Chinese cabbage under LCa stress. The scale bars in (f) and (g) are 500 μm. LCa, low‐calcium treatment (Ca^2+^ 0.1 mmol/L); MCa, medium‐calcium treatment (Ca^2+^ 5.0 mmol/L).

Through phenotypic analysis of ‘HK‐8’ Chinese cabbage at the four‐leaf stage under medium‐calcium (MCa) and low‐calcium (LCa) conditions, we observed that on the 7th day, plant phenotypes under both conditions were similar (Figure 1a). However, by the 14th day, the inner leaves of LCa‐treated plants exhibited wilting and chlorosis, while MCa‐treated plants appeared normal (Figure 1a). By the 21st day, LCa‐treated plants were significantly shorter, with inner leaves displaying yellowing, scorching and necrosis—hallmarks of tipburn symptoms (Figure 1a). Under LCa stress, the fresh and dry weights of the inner leaves and root systems were significantly reduced compared to those under MCa conditions, decreasing by 65.40% and 50.15% for inner leaves, and by 48.71% and 28.00% for roots, respectively (Figure 1b,c). Although root length remained comparable, the height of the above‐ground portion in LCa‐treated plants was significantly diminished by 45.37% (Figure 1d). Under LCa conditions, the Ca^2+^ concentration in the root system was similar to that under MCa conditions, but it decreased significantly in the outer and inner leaves by 29.40% and 40.78%, respectively (Figure 1e). The inner leaves of MCa‐treated plants displayed clear vascular bundle sheaths with regularly shaped, uniformly sized parenchyma cells (Figure 1f), whereas LCa conditions resulted in severe parenchyma cell necrosis in the leaves (Figure 1g). In summary, LCa stress suppressed Ca^2+^ uptake and plant growth in Chinese cabbage, causing cellular damage and necrosis, ultimately leading to tipburn.

### Transcriptomic analysis reveals the involvement of hormone signalling pathways in Chinese cabbage in response to LCa

**Figure 2:**
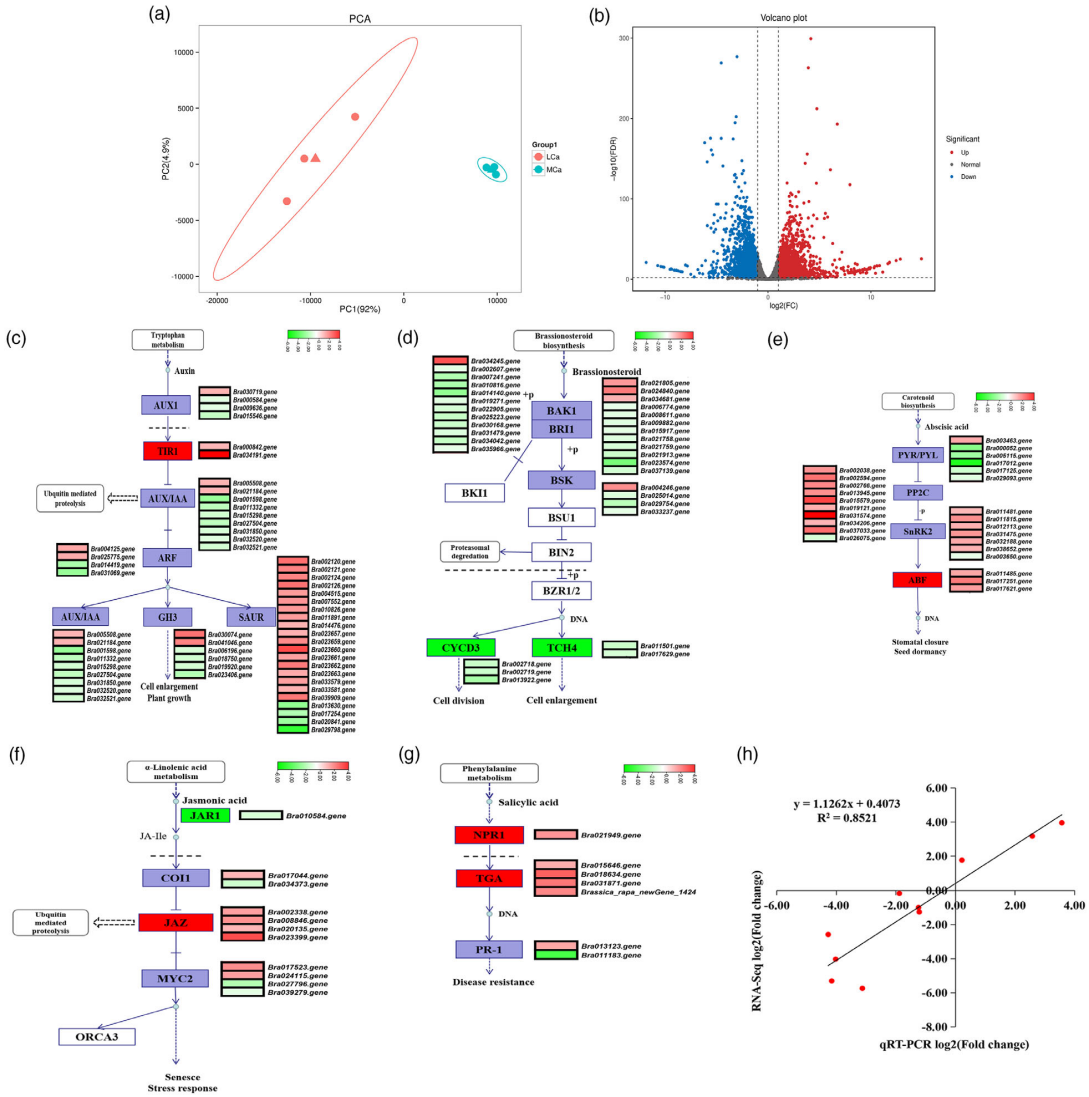
Transcriptomic data analysis of the inner leaves of Chinese cabbage under MCa and LCa treatments. (a) Principal component analysis of all genes in the inner leaves of Chinese cabbage under MCa and LCa treatments. (b) Volcano plot of differentially expressed genes (DEGs). (c) Analysis of DEGs involved in auxin signalling pathway under LCa stress. (d) Analysis of DEGs involved in brassinosteroid signalling pathway under LCa stress. (e) Analysis of DEGs involved in abscisic acid signalling pathway under LCa stress. (f) Analysis of DEGs involved in jasmonic acid signalling pathway under LCa stress. (g) Analysis of DEGs involved in salicylic acid signalling pathway under LCa stress. (h) Correlation analysis between FPKM values of 10 DEGs and qRT‐PCR data, where ‘R’ represents the Pearson correlation coefficient.

This study performed transcriptomic sequencing on the inner leaves of Chinese cabbage under medium‐calcium (MCa) and low‐calcium (LCa) conditions. A total of 42.76 Gb of clean data was obtained from the six samples (Table S3), and principal component analysis indicated that samples under each condition clustered together, demonstrating good reproducibility (Figure 2a). Using MCa conditions as the control, this study identified 5041 differentially expressed genes (DEGs) under LCa conditions, including 2574 up‐regulated and 2467 down‐regulated genes (Figure 2b). Gene Ontology (GO) analysis showed that these DEGs are primarily associated with ‘cellular process’, ‘cell’ and ‘binding’ functions within the biological process category (Figure S1a). Kyoto Encyclopedia of Genes and Genomes (KEGG) pathway and enrichment analyses revealed that these DEGs are predominantly enriched in the plant hormone signal transduction (ko04075), carbon metabolism (ko01200) and biosynthesis of amino acids (ko01230) pathways (Figure S1b,c). In the auxin signalling pathway, most genes encoding AUX1, auxin/indole‐3‐acetic acid (AUX/IAA), ARF and Gretchen Hagen 3 (GH3) were significantly down‐regulated under LCa stress (Figure 2c). In the brassinosteroid signalling pathway, most genes encoding BAK1, BRI1 and BSK proteins were also significantly down‐regulated under LCa stress, as were the three *CYCD3* and two *TCH4* genes, which are directly involved in regulating cell division and enlargement (Figure 2d). In contrast to the auxin and brassinosteroid signalling pathways, most DEGs in the abscisic acid, jasmonic acid and salicylic acid signalling pathways were significantly up‐regulated by LCa stress (Figure 2e–g). For example, in the abscisic acid signalling pathway, nine *PP2C*, six *SnRK2* and three *ABF* genes were significantly up‐regulated under LCa conditions, and these genes contribute to leaf stomatal conductance (Figure 2e). In the jasmonic acid signalling pathway, all four *JAZ* genes were significantly up‐regulated under LCa conditions (Figure 2f). In the salicylic acid signalling pathway, one *NPR1* gene, four *TGA* genes and one *PR1* gene were significantly up‐regulated under LCa conditions (Figure 2g). To validate the accuracy of the transcriptomic sequencing results, we randomly selected 10 genes and evaluated the correlation between their transcriptomic and qRT‐PCR data. The results demonstrated a Pearson's correlation coefficient squared (*R* ^2^) of 0.8521, indicating a strong positive correlation between the RNA sequencing and qRT‐PCR data (Figure 2h; Figure S2; Table S2). These findings suggest that under LCa stress, plant hormone signalling pathway genes in Chinese cabbage are activated, potentially contributing to resistance against LCa‐induced tipburn.

### Calcium signalling pathway genes *BrCNGC12* and *BrCNGC16* are key candidate genes for resistance to tipburn

**Figure 3:**
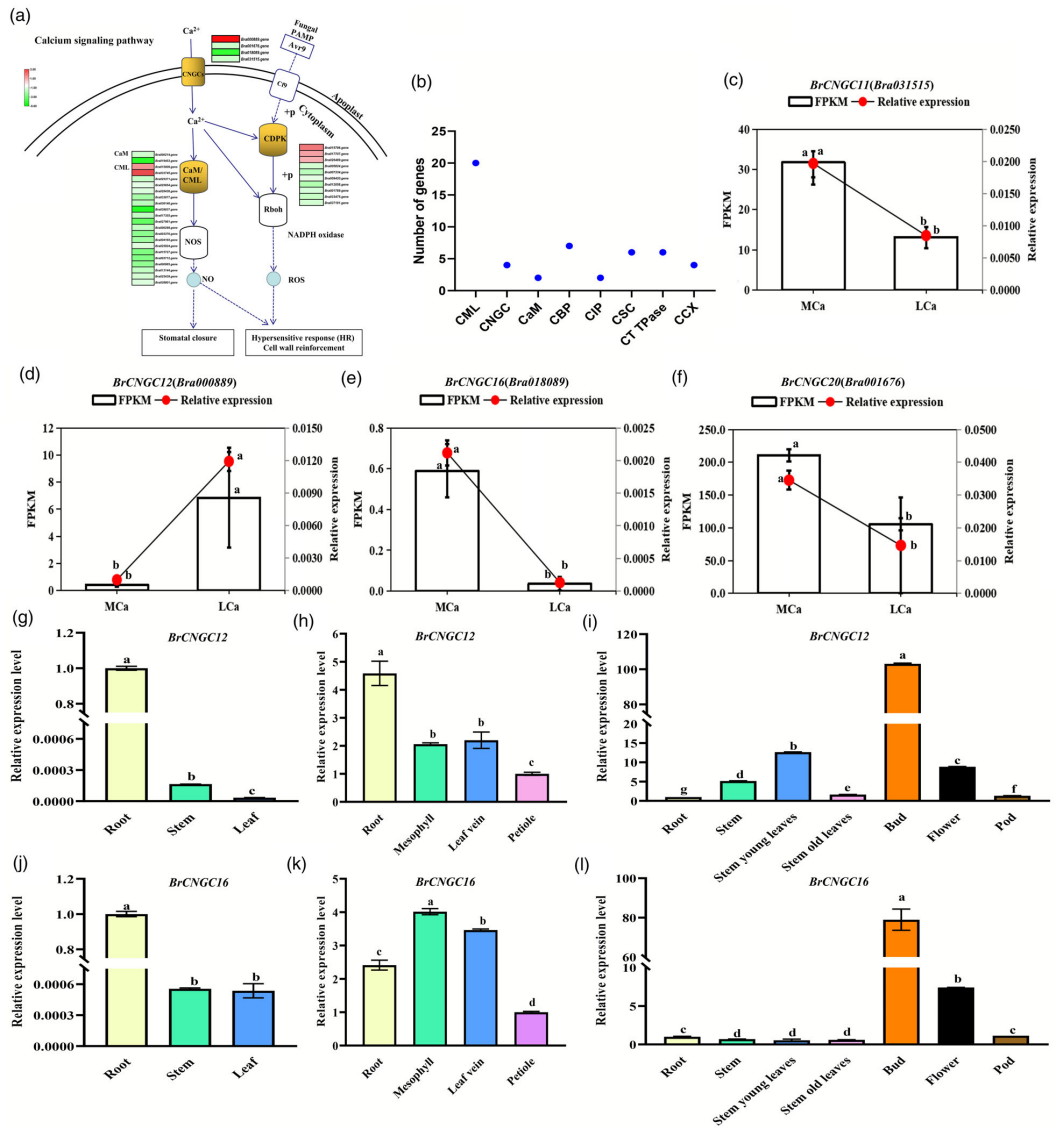
Response of Ca^2+^ absorption and transduction pathway genes to LCa stress and tissue expression patterns of *BrCNGC12*/*16*. (a) DEGs involved in the calcium signalling pathway under LCa stress. (b) Statistics of the number of major Ca^2+^ absorption‐related genes among DEGs. (c) Transcript levels and qRT‐PCR results of *BrCNGC11* under MCa and LCa treatments. (d) Transcript levels and qRT‐PCR results of *BrCNGC12* under MCa and LCa treatments. (e) Transcript levels and qRT‐PCR results of *BrCNGC16* under MCa and LCa treatments. (f) Transcript levels and qRT‐PCR results of *BrCNGC20* under MCa and LCa treatments. (g) Relative expression of *BrCNGC12* in the roots, stems and leaves of Chinese cabbage at the seedling stage. (h) Relative expression of *BrCNGC12* in the roots, leaf blades, veins and petioles of Chinese cabbage at the heading stage. (i) Relative expression of *BrCNGC12* in the roots, stems, old stem leaves, new stem leaves, flowers, pods and buds of Chinese cabbage at the early podding stage. (j) Relative expression of *BrCNGC16* in the roots, stems and leaves of Chinese cabbage at the seedling stage. (k) Relative expression of *BrCNGC16* in the roots, leaf blades, veins and petioles of Chinese cabbage at the heading stage. (l) Relative expression of *BrCNGC16* in the roots, stems, old stem leaves, new stem leaves, flowers, pods and buds of Chinese cabbage at the early podding stage. Data represent the mean of three biological replicates and three technical replicates, with error bars indicating the standard deviation (SD) of the mean. Different letters above the bars indicate significant differences (*P* < 0.05).

Through functional annotation of differentially expressed genes (DEGs), we identified 61 Ca^2+^‐related genes (Table S4), including 36 calcium signalling pathway genes. These include 20 probable calcium‐binding proteins (*CMLs*), 7 calcium‐dependent protein kinases (*CDPKs*), 4 cyclic nucleotide‐gated ion channels (*CNGCs*) and 2 calmodulins (*CaMs*), among others (Figure 3a,b). Under LCa stress, we found that three of the four *BrCNGC* genes were significantly down‐regulated, while one was significantly up‐regulated (Figure 3c–f). For example, the transcript levels of *BrCNGC12* and *BrCNGC16* under MCa conditions were 0.06 and 12.79 times those under LCa conditions, respectively, aligning closely with qRT‐PCR results (Figure 3c–f). These findings suggest that *BrCNGC12* and *BrCNGC16* may play pivotal roles in the uptake and translocation of Ca^2+^.

To investigate tissue‐specific expression differences between *BrCNGC12* and *BrCNGC16*, qRT‐PCR analysis revealed that both genes were predominantly expressed in the roots during the seedling stage. For instance, the expression level of *BrCNGC12* in roots was 21.74 times higher than that in stems and 57.14 times higher than that in leaves (Figure 3g), while *BrCNGC16* expression in roots was 2000 times higher than that in stems and leaves (Figure 3j). During the heading stage, *BrCNGC12* and *BrCNGC16* exhibited the lowest expression in petioles, with *BrCNGC12* showing the highest expression in roots (Figure 3h), and *BrCNGC16* being primarily expressed in leaf mesophyll and veins (Figure 3k). At the early podding stage, both *BrCNGC12* and *BrCNGC16* were predominantly expressed in buds, followed by flowers, with lower levels in pods and other vegetative tissues (Figure 3i,l). Given that roots and veins are crucial for nutrient uptake and translocation in Chinese cabbage during vegetative growth and that flower and bud development during the reproductive stage requires substantial Ca^2+^, these results indicate that *BrCNGC12* and *BrCNGC16* may contribute significantly to Ca^2+^ uptake and translocation, with both shared and distinct roles.

### *BrCNGC12* negatively regulates the resistance to tipburn in Chinese cabbage

**Figure 4:**
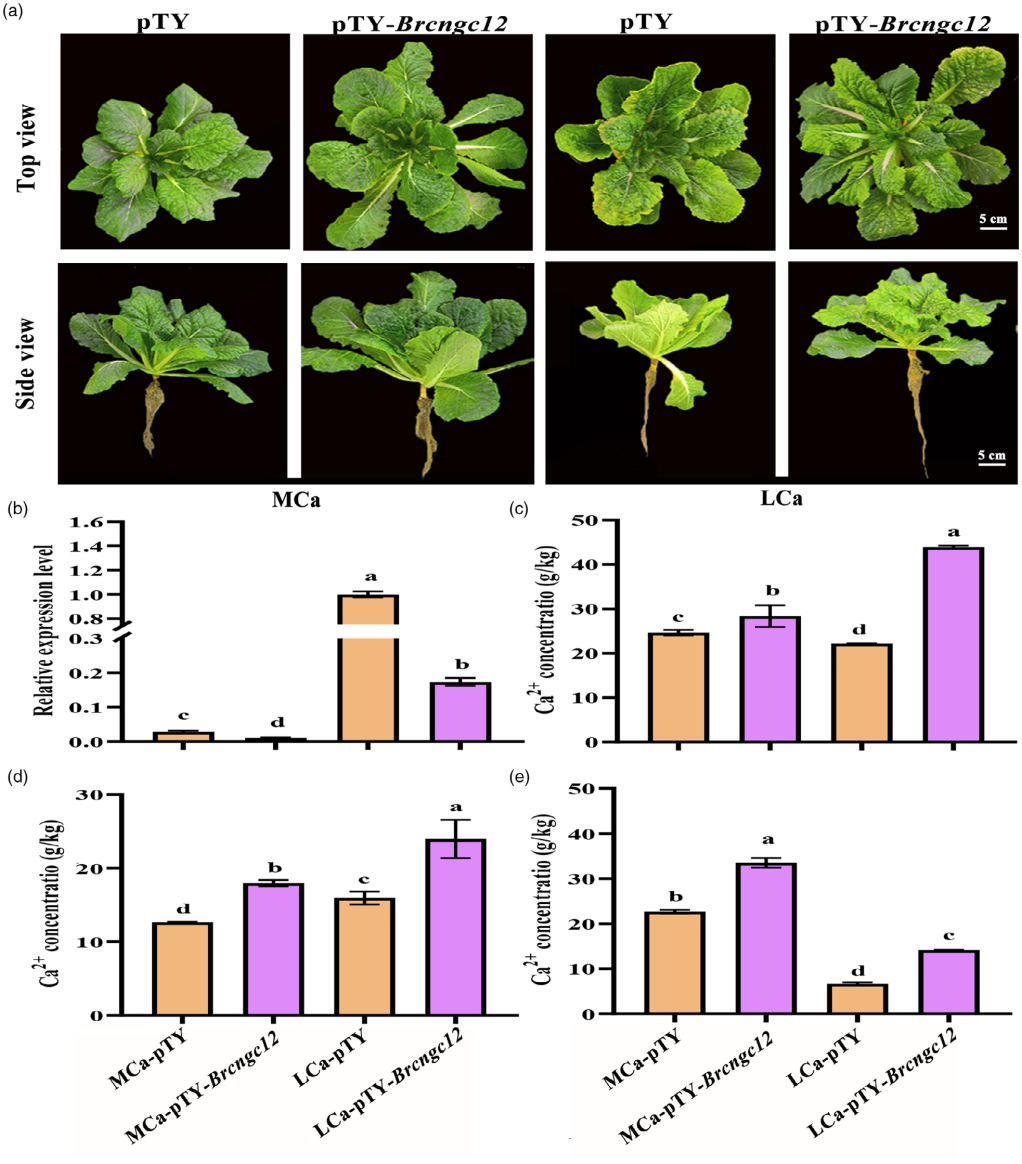
Response of pTY‐*Brcngc12* and pTY plants to LCa stress. (a) Phenotypes of pTY‐*Brcngc12* and pTY plants under MCa and LCa treatments; (b) Relative expression levels of *BrCNGC12* in pTY‐*Brcngc12* and pTY plants under MCa and LCa treatments. (c) Ca^2+^ concentrations in the outer leaves of pTY‐*Brcngc12* and pTY plants under MCa and LCa treatments. (d) Ca^2+^ concentrations in the inner leaves of pTY‐*Brcngc12* and pTY plants under MCa and LCa treatments. (e) Ca^2+^ concentrations in the roots of pTY‐*Brcngc12* and pTY plants under MCa and LCa treatments. Data represent the mean of three biological replicates and three technical replicates, with error bars indicating the standard deviation (SD) of the mean. Different letters above the bars indicate significant differences (*P* < 0.05).

To examine the response of *BrCNGC12* to LCa stress, this study first cloned its open reading frame sequence, which comprises 1185 bp and encodes 394 amino acids (Figure S3). The theoretical isoelectric point (*pI*) of the BrCNGC12 protein is 9.01, with a relative molecular weight (Mw) of 45545.13 Da. It consists of 61.68% alpha‐helices, 24.62% random coils, 12.18% extended strands and 1.52% beta‐turns (Figure S4a). The BrCNGC12 protein was predicted to be a transmembrane protein harbouring a conserved Ion_trans domain (Figures S3 and S4b). The *BrCNGC12* gene was subsequently silenced using virus‐induced gene silencing (VIGS) technology. Phenotypic analysis showed that under LCa conditions, control plants (pTY) exhibited prominent tipburn at the leaf margins compared to MCa conditions, whereas *Brcngc12*‐silenced plants (pTY‐*Brcngc12*) displayed no significant phenotypic differences relative to MCa conditions (Figure 4a). qRT‐PCR analysis revealed that the expression level of *BrCNGC12* in pTY‐*Brcngc12* plants was significantly lower than in pTY plants under MCa conditions (Figure 4b). However, LCa stress significantly up‐regulated *BrCNGC12* expression, with its level in pTY plants being 5.76 times higher than that in pTY‐*Brcngc12* plants (Figure 4b). Further analysis of Ca^2+^ concentrations indicated that LCa conditions increased Ca^2+^ content in the inner leaves (Figure 4d) while reducing it in the outer leaves and roots of Chinese cabbage (Figure 4c,e). Moreover, under LCa stress, the Ca^2+^ content in the outer leaves, inner leaves and roots of pTY‐*Brcngc12* plants was 1.97, 1.50 and 2.11 times higher than that in pTY plants, respectively (Figure 4c–e). These results demonstrate that *BrCNGC12* is up‐regulated by LCa stress and, when silenced in Chinese cabbage, enhances Ca^2+^ uptake and translocation, thereby increasing resistance to tipburn.

### Heterologous expression of *BrCNGC16* enhances *A. thaliana* resistance to tipburn

**Figure 5:**
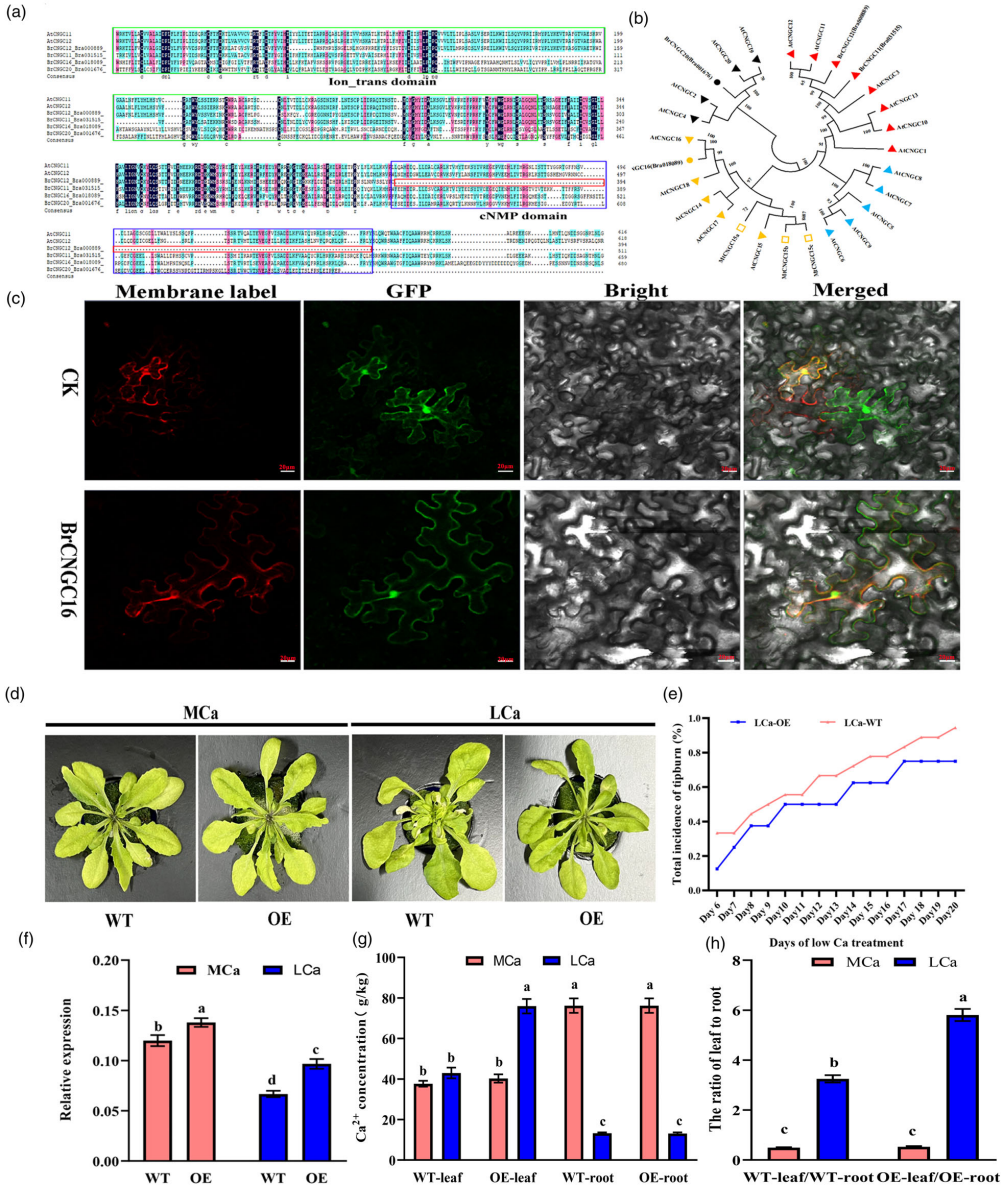
Bioinformatics analysis of *BrCNGC16* and the response of transgenic *A. thaliana* to LCa stress. (a) Sequence alignment and conserved domain analysis of BrCNGC12, BrCNGC16 and other CNGC proteins. (b) Phylogenetic relationship of BrCNGC12, BrCNGC16 with CNGC proteins from other species. The phylogenetic tree was constructed using the Neighbour‐Joining method with MEGA6.0 software. Bootstrap values higher than 97% are shown at the nodes. (c) Subcellular localization analysis of BrCNGC16 in tobacco. Membrane label: mCherry, pm‐rbCD3‐1008; red fluorescence is observed only on the cell membrane and nuclear membrane; GFP, green fluorescence image of tobacco leaf cells; Merged, overlay of red fluorescence, green fluorescence and bright‐field image; scale bar: 20 μm. (d) Phenotypes of *BrCNGC16*‐transgenic *A. thaliana* and WT plants under MCa and LCa treatments. (e) Statistics of the total incidence of tipburn in transgenic and WT plants under LCa treatment. (f) Relative expression level analysis of *BrCNGC16* in transgenic and WT plants under MCa and LCa treatments. (g) Ca^2+^ concentrations in the roots and leaves of transgenic and WT plants. (h) Ratio of Ca^2+^ concentration in leaves to roots of transgenic and WT plants under MCa and LCa treatments. Data represent the mean of three biological replicates and three technical replicates, with error bars indicating the standard deviation (SD) of the mean. Bars with different letters indicate significant differences (*P* < 0.05).

Given that *BrCNGC16* responds to LCa stress and exhibits an opposite expression trend to *BrCNGC12* (Figure 3), we conducted further analysis of *BrCNGC16* in this study. The open reading frame of *BrCNGC16* was cloned, revealing that it comprises 2121 bp and encodes 706 amino acids (Figure 5a). Analysis using SMART software predicted that BrCNGC16 harbours a conserved Ion_trans domain at the N‐terminal region (amino acids 53–387) and a cyclic nucleotide‐monophosphate binding (cNMP) domain at the C‐terminus, the latter absent in BrCNGC12 (Figure 5a). Phylogenetic analysis showed that BrCNGC16 clusters with AtCNGC16, AtCNGC18, AtCNGC14 and AtCNGC17 in one evolutionary branch, while BrCNGC12 clusters with AtCNGC12 in another (Figure 5b; Table S5), suggesting potential functional divergence between BrCNGC16 and BrCNGC12. Subcellular localization demonstrated that a GFP fusion protein with BrCNGC16 emitted green fluorescence at the plasma membrane, co‐localizing with the red fluorescence of a membrane‐tagged protein (Figure 5c) and also showed green fluorescence in the nucleus, fully overlapping with the nuclear membrane (Figure 5c). This indicates that the BrCNGC16 protein likely functions at both the plasma membrane and within the nucleus.

To explore the role of *BrCNGC16* in tipburn resistance, we screened 60 T3‐generation *A. thaliana* plants heterologously expressing *BrCNGC16* (from five lines) using hygromycin resistance gene selection and *β*‐glucuronidase (GUS) histochemical staining (Figure S5). Phenotypic analysis revealed no significant differences between transgenic and wild‐type (WT) plants under MCa conditions. However, under LCa conditions, WT plants exhibited tipburn symptoms, whereas transgenic *A. thaliana* plants displayed resistance to tipburn (Figure 5d). The incidence of tipburn in transgenic plants was significantly lower than in WT plants throughout the LCa treatment period (Figure 5e). qRT‐PCR analysis indicated that *BrCNGC16* expression was significantly down‐regulated under LCa conditions (Figure 5f). While *BrCNGC16* had minimal impact on root Ca^2+^ content, the leaf Ca^2+^ concentration in transgenic plants was 2.01 times higher than that in WT plants (Figure 5g). Further analysis showed that the leaf‐to‐root Ca^2+^ concentration ratio in transgenic plants was 1.8 times higher than that in WT plants under LCa stress (Figure 5h), suggesting that *BrCNGC16* plays a key role in facilitating Ca^2+^ uptake and translocation from roots to leaves in *A. thaliana* under LCa stress.

### Partial silencing of *BrCNGC16* reduces the resistance of Chinese cabbage to LCa‐induced tipburn

**Figure 6:**
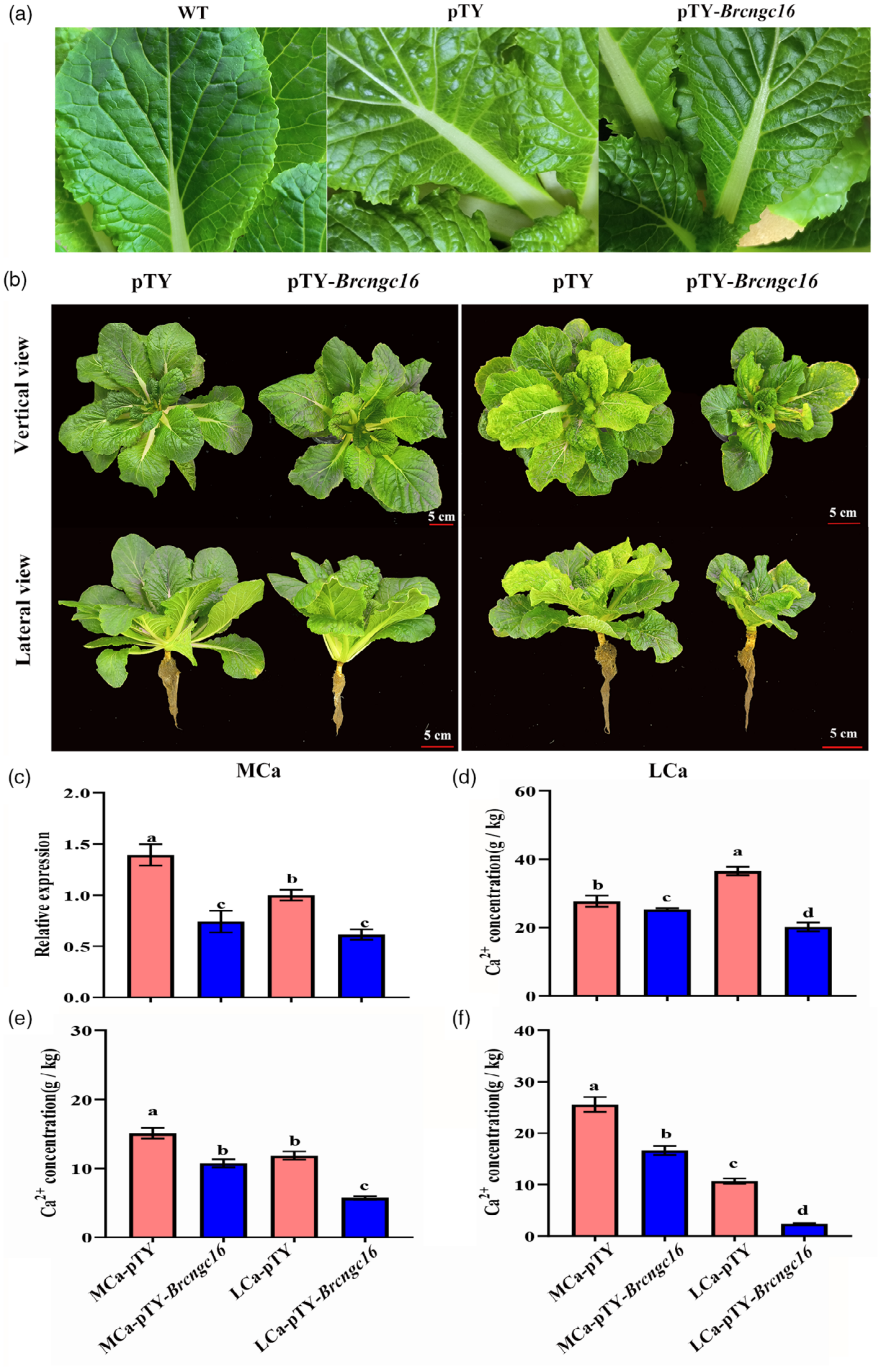
Response of pTY‐*BrCNGC16* and pTY plants to LCa stress. (a) Local phenotypes of pTY‐*Brcngc16* and pTY plants 20 days after viral inoculation. (b) Phenotypes of pTY‐*Brcngc16* and pTY plants under MCa and LCa treatments. (c) Relative expression levels of *BrCNGC16* in the inner leaves of pTY‐*Brcngc16* and pTY plants under MCa and LCa treatments. (d) Ca^2+^ concentrations in the outer leaves of pTY‐*Brcngc16* and pTY plants under MCa and LCa treatments. (e) Ca^2+^ concentrations in the inner leaves of pTY‐*Brcngc16* and pTY plants under MCa and LCa treatments. (f) Ca^2+^ concentrations in the roots of pTY‐*Brcngc16* and pTY plants under MCa and LCa treatments. Data represent the mean of three biological replicates and three technical replicates, with error bars indicating the standard deviation (SD) of the mean. Bars with different letters indicate significant differences (*P* < 0.05).

This study utilized virus‐induced gene silencing (VIGS) to investigate the role of *BrCNGC16* in Chinese cabbage resistance to LCa‐induced tipburn. Twenty days post‐inoculation, the leaves of *Brcngc16*‐silenced plants (pTY‐*Brcngc16*) and control plants (pTY) exhibited significant chlorosis compared to wild‐type (WT) plants, displaying typical symptoms of turnip yellow mosaic virus infection (Figure 6a). Based on these traits, we obtained six pTY‐*Brcngc16* and six pTY plants. Under MCa conditions, no significant differences were observed in the above‐ground portions of pTY‐*Brcngc16* and pTY plants, though pTY‐*Brcngc16* showed weaker roots compared to pTY (Figure 6b). Under LCa conditions, pTY‐*Brcngc16* plants displayed more severe tipburn symptoms, smaller plant size and underdeveloped roots relative to pTY (Figure 6b). qRT‐PCR analysis revealed that *BrCNGC16* expression in the inner leaves of pTY‐*Brcngc16* was 0.53 times lower than that in pTY under MCa conditions, confirming successful silencing, with LCa conditions further significantly suppressing *BrCNGC16* expression (Figure 6c). In pTY plants, LCa conditions elevated Ca^2+^ content in the outer leaves, but this content was only 0.55 times that in pTY‐*Brcngc16* (Figure 6d). In the inner leaves, the Ca^2+^ concentration in pTY‐*Brcngc16* under LCa conditions was 0.48 times that in pTY (Figure 6e). In roots, the Ca^2+^ concentration in pTY‐*Brcngc16* under LCa conditions was 0.23 times that in pTY (Figure 6f). These findings indicate that silencing *BrCNGC16* impairs Ca^2+^ uptake in Chinese cabbage, thereby reducing resistance to tipburn.

### Positive identification and Ca^2+^ content analysis of overexpressed *BrCNGC16* in Chinese cabbage

**Figure 7:**
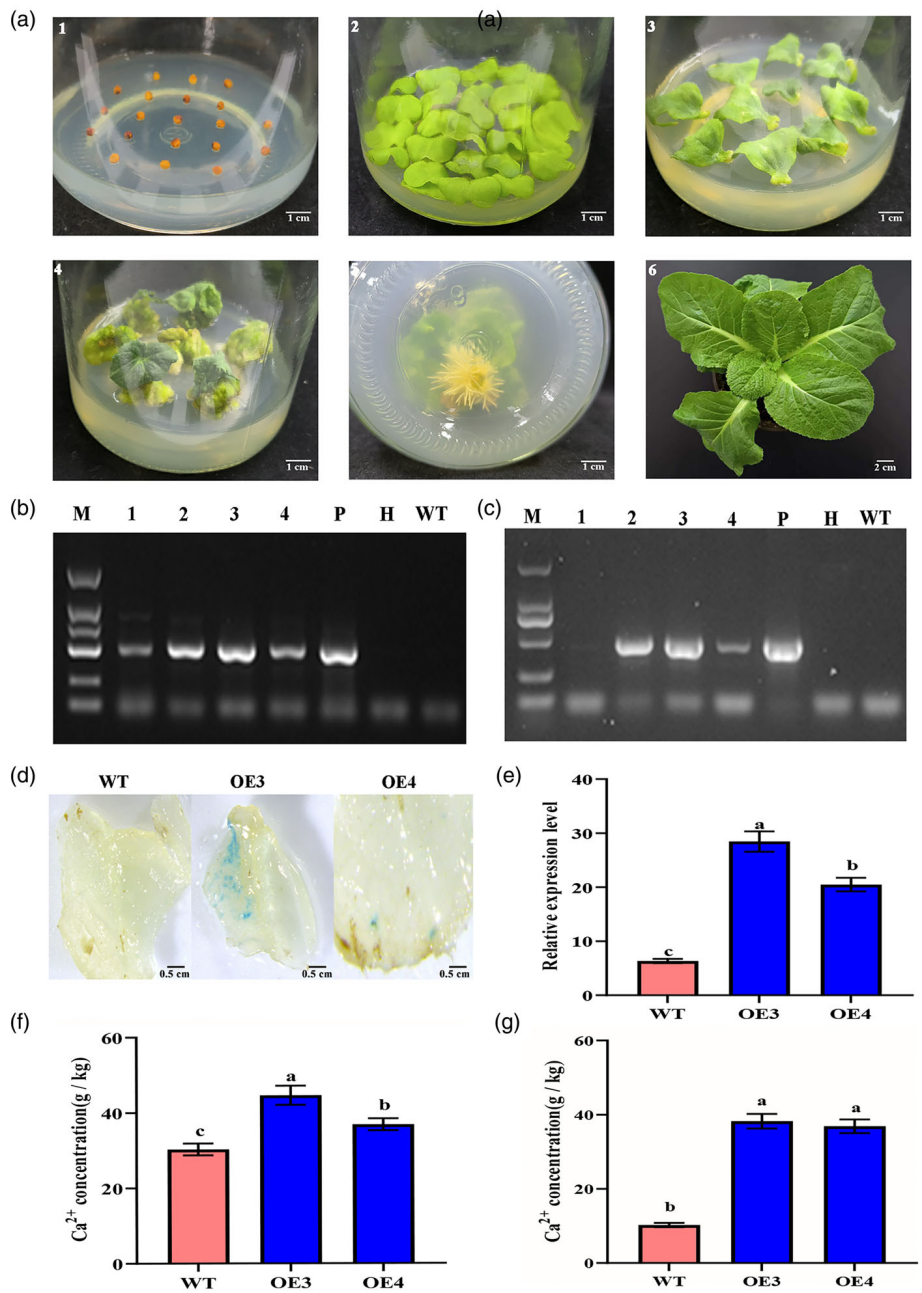
Positive validation of *BrCNGC16*‐overexpressing Chinese cabbage plants and their involvement in Ca^2+^ uptake. (a) Genetic transformation of *BrCNGC16* in Chinese cabbage plants. 1, seed sowing; 2, pre‐culture; 3, co‐culture; 4, bud induction; 5, rooting induction; 6, plantlet formation. (b) Hygromycin resistance gene detection, with a band size of 481 bp. (c) Target gene detection, with an amplified band size of 463 bp, including 383 bp from the 3′ end of the target gene and 80 bp from the vector sequence; Marker (M) is 1000 bp. From bottom to top: 100, 250, 500, 750, 1000, 2000 bp; P represents plasmid (positive control), WT represents the gene detection using wild‐type Chinese cabbage DNA (negative control), H represents water (negative control), and 1–4 represent gene detection using DNA from tissue‐cultured plants. (d) GUS histochemical staining. (e) Relative expression of *BrCNGC16* gene in *BrCNGC16*‐overexpressing and WT Chinese cabbage leaf tissues (qRT‐PCR). (f) Ca^2+^ content in the outer leaves of *BrCNGC16*‐overexpressing and WT Chinese cabbage plants. (g) Ca^2+^ content in the inner leaves of *BrCNGC16*‐overexpressing and WT Chinese cabbage plants. Data represent the mean of three biological replicates and three technical replicates, with error bars indicating the standard deviation (SD) of the mean. Bars with different letters indicate significant differences (*P* < 0.05).

This study employed *Agrobacterium*‐mediated genetic transformation to generate 13 *BrCNGC16*‐overexpressing plants. Figure 7a illustrates the various stages of tissue culture, with the media used detailed in Table 1. To confirm positive Chinese cabbage plants, DNA from the leaves of T2‐generation plants was used as a template to detect the *hygromycin resistance* (*Hyg* ^*r*^) gene via PCR. Results showed that this gene was absent in wild‐type (WT) plants but present in both the positive seedlings and the recombinant plasmid pTCK303‐*BrCNGC16* (P) (Figure 7b). Further PCR detection of the target gene revealed that it was amplified in most plants containing the *Hyg* ^*r*^ gene (Figure 7c). GUS histochemical staining demonstrated that WT plant leaves remained unstained, whereas transgenic plant leaves stained blue (Figure 7d). qRT‐PCR analysis indicated that the relative expression level of *BrCNGC16* in the leaves of transgenic plants was significantly higher than that in WT plants (Figure 7e). The Ca^2+^ content in the outer leaves of *BrCNGC16*‐overexpressing plants was 1.3–1.47 times higher than that in control plants (Figure 7f), while in the inner leaves, it was 3.60–3.73 times higher than that in control plants (Figure 7g). These findings suggest that overexpression of *BrCNGC16* enhances Ca^2+^ uptake in both outer and inner leaves, with greater efficiency in the inner leaves.

**Table 1:** The list of culture medium components used for the genetic transformation of *BrCNGC16* in Chinese cabbage

Culture medium	Components of the medium	pH value
Germination medium	1/2 MS + 20 g/L Sucrose + 7 g/L Agar powder	5.8
Pre‐culture	MS + 20 g/L Sucrose + 7 g/L Agar powder + 0.5 mg/L NAA + 4 mg/L 6‐BA + 4 mg/L AgNO_3_	5.8
Maceration liquor	MS + 100 umol/L AS + 20 g/L Sucrose	5.2
Co‐culture	MS + 20 g/L Sucrose + 7 g/L Agar powder + 0.5 mg/L NAA + 4 mg/L 6‐BA + 4 mg/L AgNO_3_ + 100 μmol/L AS	5.8
Screening and bud induction medium	MS + 20 g/L Sucrose + 7 g/L Agar powder + 0.5 mg/L NAA + 4 mg/L 6‐BA + 4 mg/L AgNO_3_ + 200 mg/L Cef + 100 mg/L Car + 20 mg/L Hyg	5.8
Rooting medium	MS + 20 g/L Sucrose + 7 g/L Agar powder + 1 mg/L NAA + 200 mg/L Cef + 100 mg/L Car	5.8

### Overexpression of *BrCNGC16* enhances Ca^2+^ uptake and improves the resistance to tipburn in Chinese cabbage

**Figure 8:**
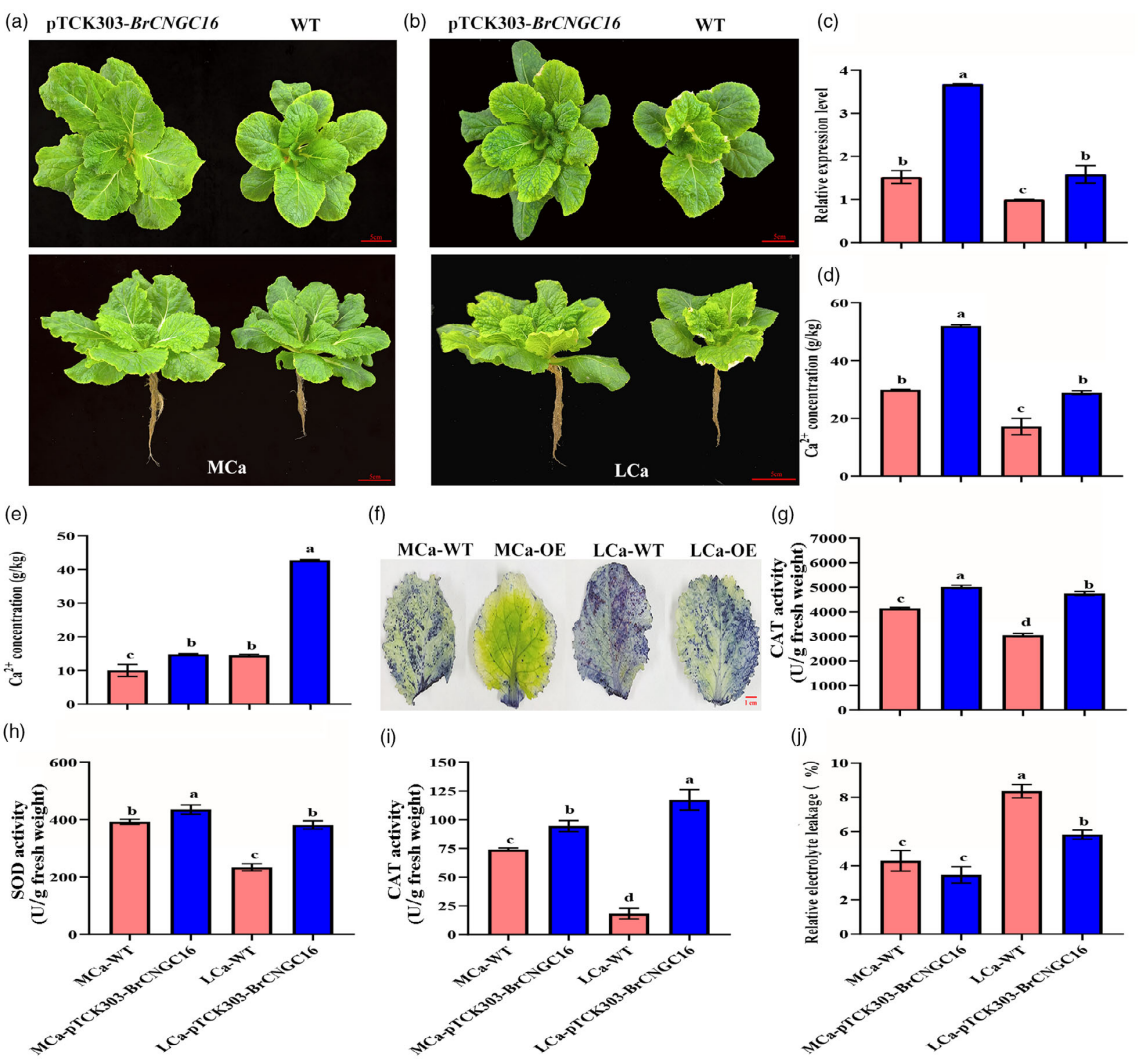
Analysis of phenotype and physiological indices in *BrCNGC16* overexpressing Chinese cabbage under LCa stress. (a) Phenotype of pTCK303‐*BrCNGC16* and WT plants under MCa treatment. (b) Phenotype of pTCK303‐*BrCNGC16* and WT plants under LCa treatment. (c) Analysis of *BrCNGC16* expression in pTCK303‐*BrCNGC16* and WT plants under MCa and LCa treatments. (d) Ca^2+^ concentration in the inner leaves of pTCK303‐*BrCNGC16* and WT plants under MCa and LCa treatments. (e) Ca^2+^ concentration in the roots of pTCK303‐*BrCNGC16* and WT plants under MCa and LCa treatments. (f) NBT staining in the leaves of pTCK303‐*BrCNGC16* and WT plants under MCa and LCa treatments. (g) POD activity in pTCK303‐*BrCNGC16* and WT plants under MCa and LCa treatments. (h) SOD activity in pTCK303‐*BrCNGC16* and WT plants under MCa and LCa treatments. (i) CAT activity in pTCK303‐*BrCNGC16* and WT plants under MCa and LCa treatments. (j) Electrolyte leakage in the leaves of pTCK303‐*BrCNGC16* and WT plants under MCa and LCa treatments. Data represent the mean of three biological replicates and three technical replicates, with error bars indicating the standard deviation (SD) of the mean. Bars with different letters indicate significant differences (*P* < 0.05).

To assess the response of *BrCNGC16* to LCa stress, T2‐generation *BrCNGC16*‐overexpressing Chinese cabbage and WT plants were subjected to MCa and LCa conditions (Figure 8). Phenotypic observations revealed that pTCK303‐*BrCNGC16* plants exhibited larger plant size and a more developed root system relative to WT plants under MCa conditions (Figure 8a). Under LCa conditions, transgenic plants demonstrated greater resistance to tipburn compared to WT plants (Figure 8b). qRT‐PCR analysis showed that LCa stress suppressed *BrCNGC16* expression, with the expression level in the leaves of transgenic plants under LCa stress being 1.58 times higher than that in control plants (Figure 8c). Ca^2+^ concentration analysis indicated that LCa conditions reduced Ca^2+^ concentration in the inner leaves, yet the Ca^2+^ concentration in transgenic plants was 1.82 times higher than that in WT plants (Figure 8d). In roots, the Ca^2+^ concentration in transgenic plants under LCa conditions was 2.95 times higher than that in WT plants (Figure 8e). These results demonstrate that *BrCNGC16* bolsters tipburn resistance under LCa stress by increasing plant Ca^2+^ concentrations.

Furthermore, NBT histochemical staining revealed that transgenic plant leaves exhibited lighter staining under LCa conditions compared to WT plants (Figure 8f), suggesting that *BrCNGC16* may contribute to scavenging superoxide anions. Analysis of antioxidant enzyme activities in the leaves of transgenic and WT plants under MCa and LCa conditions showed that under LCa conditions, POD activity in pTCK303‐*BrCNGC16* plants was 1.56 times higher than that in WT plants, SOD activity was 1.63 times higher, and CAT activity was 6.41 times higher (Figure 8g–i), indicating a greater antioxidant capacity. Measurements of relative electrical conductivity in plant leaves under MCa and LCa conditions revealed no significant difference between WT and pTCK303‐*BrCNGC16* plants under MCa conditions, whereas under LCa conditions, the electrical conductivity of WT plants was 1.44 times higher than that of pTCK303‐*BrCNGC16* plants (Figure 8j). These findings indicate that overexpression of *BrCNGC16* under LCa conditions mitigates excessive reactive oxygen species (ROS) accumulation in Chinese cabbage, thereby enhancing tipburn resistance.

## Discussion

Ca^2+^ is essential for plant growth and development, contributing to multiple biological processes (Lambers *et al*., 2008; Marschner, 1995; Reddy, 2001). First, Ca^2+^ is a key component of pectin in the cell wall, supporting its structure and function, maintaining elasticity and toughness, and facilitating cell division and elongation (Lambers *et al*., 2008). Moreover, Ca^2+^ acts as an activator or inhibitor of numerous enzymes, participating in various metabolic processes—such as photosynthesis, respiration and nitrogen and carbohydrate metabolism—thereby influencing plant growth and physiological functions (Marschner, 1995). Additionally, as a critical intracellular signalling molecule, Ca^2+^ plays a significant role in responses to environmental stresses, markedly affecting processes like stomatal regulation, hormone signalling and cell death (Reddy, 2001). Tipburn, a prevalent physiological disorder in Chinese cabbage, arises from multiple factors, with Ca^2+^ deficiency being one of the most critical (Zhang *et al*., 2021). When the roots of Chinese cabbage exhibit reduced Ca^2+^ uptake, the plant fails to acquire sufficient Ca^2+^, triggering tipburn (Zhang *et al*., 2021). Given that Chinese cabbage is a widely cultivated and popular vegetable, enhancing its capacity to absorb and translocate Ca^2+^ is a key strategy for achieving high‐yield, high‐quality crops.

By subjecting Chinese cabbage seedlings to Ca^2+^ deficiency, this study observed that plants showed no symptoms of Ca^2+^ deficiency by the 7th day of LCa conditions. However, from the 14th day of LCa conditions, seedlings began displaying symptoms such as wilting and chlorosis in the inner leaves. By the 21st day, plants exhibited yellowing, scorching and necrosis, consistent with the previously reported progression of tipburn in Chinese cabbage (Kuo *et al*., 1981; Yuan *et al*., 2021) and characteristic of typical tipburn symptoms. The study further revealed that LCa stress suppresses growth and development indicators, reduces Ca^2+^ content and disrupts cell structure in Chinese cabbage seedlings (Figure 1), aligning with findings by Zain *et al*. (2022). Notably, under LCa stress, the Ca^2+^ content in inner leaves decreased more significantly than in outer leaves, supporting the conclusion that LCa stress predominantly affects inner leaves (Yuan *et al*., 2021). Literature indicates that LCa stress also causes stunted growth, leaf yellowing and wilting, and impairs photosynthesis, water metabolism and stress tolerance in plants (Abd_Allah *et al*., 2017; Dolatabadian *et al*., 2013). Thus, bolstering resistance to Ca^2+^ deficiency‐induced tipburn in Chinese cabbage is of considerable importance.

The molecular mechanisms underlying tipburn resistance in Chinese cabbage remain poorly elucidated. However, evidence suggests that hormone signalling pathways are pivotal to plant growth and development, and their dysregulation may heighten stress sensitivity while reducing disease resistance and stress tolerance (Lee *et al*., 2016). For example, prior studies have demonstrated that aberrant expression of auxin signalling pathway genes can induce tipburn symptoms in Brassica oleracea (Lee *et al*., 2016). In Chinese cabbage, LCa stress down‐regulates transcription of auxin signalling pathway genes (e.g. *AUX1*, *AUX*/*IAA* and *ARF*) and brassinosteroid signalling pathway genes (e.g. *BAK1*, *BRI1* and *BSK*) (Figure 2c–d). In contrast, most differentially expressed genes (DEGs) in the abscisic acid, jasmonic acid and salicylic acid signalling pathways are significantly up‐regulated under LCa conditions (Figure 2e–g). Based on this analysis, Chinese cabbage may engage hormone signalling pathways to confer resistance to tipburn under Ca^2+^ deficiency stress, though the underlying molecular mechanisms warrant further investigation.

Under stress conditions, the calcium signalling pathway is instrumental in plant defence responses (Dodd *et al*., 2010; Qin *et al*., 2024). Cyclic nucleotide‐gated channels (CNGCs) primarily facilitate the transport of cations such as Ca^2+^, Na^+^ and K^+^ (Charpentier *et al*., 2016). Calmodulin and calmodulin‐like proteins (CaMs/CMLs) undergo conformational changes upon Ca^2+^ binding, activating a diverse array of target proteins, including calcium‐dependent protein kinases (CDPKs) and calmodulin‐binding proteins (CaBPs) (Yuan *et al*., 2021). These proteins enhance plant stress defence by elevating intracellular Ca^2+^ levels, boosting reactive oxygen species (ROS) scavenging enzyme activity, regulating stomatal closure, strengthening cell walls or modulating stress‐related gene expression (Yuan *et al*., 2021). To date, research on tipburn in Chinese cabbage has predominantly focused on proteins involved in Ca^2+^ uptake and translocation, such as calreticulin BrCRT2 and Ca^2+^/H^+^ antiporters BrCAX1‐1 and BrCAX1‐2 (Cheng *et al*., 2015; Cui *et al*., 2023; Su *et al*., 2019). Although the calcium signalling pathway is well established in plant defence, limited studies have linked it to tipburn resistance in Chinese cabbage. Through Gene Ontology (GO) and Kyoto Encyclopedia of Genes and Genomes (KEGG) analyses of DEGs from transcriptomic data of Chinese cabbage inner leaves under MCa and LCa conditions, this study identified significant responses to LCa stress among calcium signalling pathway genes, including 20 calmodulin‐like (*CMLs*), 10 *CDPKs*, 4 *CNGCs*, 2 *CaMs* and 7 calmodulin‐binding protein (*CBP*) genes (Table 1; Figure 3b). These findings affirm the association between calcium signalling pathway genes and tipburn resistance.

CNGCs are critical components of the calcium signalling pathway (Chan *et al*., 2003). Research on *AtCNGC12* in *Arabidopsis thaliana* is relatively well‐documented. For instance, *AtCNGC12* contributes to pathogen defence and programmed cell death (DeFalco *et al*., 2016). Studies in yeast have shown that *AtCNGC12* restores growth in K^+^‐ and Ca^2+^‐uptake‐deficient strains (Baxter *et al*., 2008), while in Xenopus oocytes, its expression induces Ca^2+^ translocation (Zhang *et al*., 2019). These findings suggest that *AtCNGC12* positively regulates Ca^2+^ translocation. *AtCNGC12* (Gene ID: *AT2G46450*) shares an evolutionary branch with *BrCNGC12*, yet their amino acid sequence identity is only 37.38% (Figure 5). Domain prediction reveals that *BrCNGC12* contains an Ion_trans domain, whereas *AtCNGC12* also possesses a CNBD/cNMP domain of unknown function at the C‐terminus. This suggests that while *BrCNGC12* may share some functions with *AtCNGC12*, distinct roles could arise from structural differences. Contrary to expectations, this study found that silencing *BrCNGC12* in Chinese cabbage enhanced Ca^2+^ uptake, opposing the positive regulation observed with AtCNGC12. Domain prediction revealed that BrCNGC12 lacks the C‐terminal cyclic nucleotide‐binding domain (cNMP domain) present in AtCNGC12 (Figure 5), which is critical for cyclic nucleotide‐dependent channel gating in Arabidopsis (Charpentier *et al*., 2016). This structural divergence may impair BrCNGC12's ability to transduce Ca^2+^ signals under low‐calcium conditions, leading to compensatory up‐regulation of other Ca^2+^ transporters. However, this hypothesis requires further exploration through electrophysiological characterization of BrCNGC12.

This study also examined the key candidate gene *BrCNGC16* in tipburn resistance. Subcellular localization confirmed that *BrCNGC16* is positioned at the plasma membrane and nucleus, consistent with the functional sites of Ca^2+^ channel proteins (Rahmati Ishka *et al*., 2018). Phylogenetic analysis indicated that *BrCNGC16* clusters with *AtCNGC16*, *AtCNGC18*, *AtCNGC14* and *AtCNGC17* in *A. thaliana*, suggesting potential functional similarity (Figure 5b). Although detailed reports on *AtCNGC16* function are scarce (Rahmati Ishka *et al*., 2018), elucidating *BrCNGC16* function offers valuable insights into CNGC variation across species. Functional analysis demonstrated that *A. thaliana* plants heterologously expressing *BrCNGC16* exhibited increased tipburn resistance (Figure 5). In Chinese cabbage, partial silencing of *BrCNGC16* diminished resistance, while overexpression enhanced it (Figures 6 and 8). Ca^2+^ content analysis in leaves and roots, alongside other physiological indicators, reinforced this, showing that *BrCNGC16* promotes Ca^2+^ translocation from roots to leaves, thereby bolstering resistance to Ca^2+^ deficiency‐induced tipburn. Under environmental stress, excessive reactive oxygen species (ROS) production signals plant abnormality (Ahmad *et al*., 2018). Peroxidase (POD), superoxide dismutase (SOD) and catalase (CAT) are key antioxidant enzymes in ROS scavenging (Neves *et al*., 2010). Here, under LCa stress, POD, SOD and CAT activities in leaves of *BrCNGC16*‐overexpressing plants were significantly higher than in WT plants (Figure 8), indicating that *BrCNGC16* enhances LCa stress resistance by mitigating ROS accumulation.

In summary, this study clarified transcriptomic changes in inner leaves under LCa stress, revealing that Chinese cabbage may leverage hormone and calcium signalling pathways to resist Ca^2+^ deficiency‐induced tipburn. Specifically, within the calcium signalling pathway, *BrCNGC12*, up‐regulated by LCa stress, negatively regulates resistance, while *BrCNGC16*, suppressed by LCa stress, positively regulates it. Recent studies on tomato *SlCNGC16* homologues demonstrated their role in mediating Ca^2+^ redistribution under nutrient stress, which aligns with our findings on *BrCNGC16*'s contribution to Ca^2+^ translocation (Wang *et al*., 2023). These findings provide novel insights for breeding tipburn‐resistant Chinese cabbage varieties. Future studies could explore downstream genes and regulatory networks of *BrCNGC16*, as well as its interactions with other Ca^2+^ channel proteins, to deepen our understanding of the molecular mechanisms underpinning tipburn resistance in Chinese cabbage.

## Materials and methods

### Trial materials and treatment

In this study, the tipburn‐sensitive Chinese cabbage variety ‘HK‐8’ was chosen as the experimental material. Following seed germination, seeds were sown in plug trays. At the ‘2 leaves and 1 heart’ stage [two fully expanded leaves and one developing central bud (heart leaf)], seedlings were transplanted into turnover boxes containing modified Hoagland nutrient solution for medium‐calcium (MCa) (control, 5 mmol/L) and low‐calcium (LCa) (Ca^2+^‐deficient, 0.01 mmol/L) conditions to induce tipburn. Each condition was replicated across five turnover boxes, with four seedlings per box. The cultivation process was conducted in an artificial climate chamber under the following conditions: 25 °C with 16 h of light (800 μmol/m^2^/s) and 20 °C with 8 h of darkness and a relative humidity of 65%. During cultivation, the nutrient solution fully submerged the roots and was replaced every 3 days. Growth indicators—including plant height, as well as dry and fresh weights of the above‐ground portions and roots—were recorded via photographs taken on days 7, 14 and 21 of cultivation. On the 14th day of LCa and MCa conditions, Ca^2+^ content was quantified in the outer leaves, inner leaves and roots. Additionally, inner leaves from this time point were harvested as samples for subsequent transcriptome sequencing to investigate the effects of tipburn.

### Transcriptome sequencing analysis

Chinese cabbage inner leaves treated with MCa and LCa for 14 days were used for transcriptome sequencing, with three replicates per treatment, totalling six samples. RNA was extracted using RNA‐Solv^@^ reagent (Code No. R6830‐01, Omega Bio‐Tek, Beijing, China) and assessed for purity, concentration and integrity with NanoDrop, Qubit 2.0 and Agilent 2100 (Desjardins and Conklin, 2010; Michele *et al*., 2013; Panaro *et al*., 2000). The RNA was then reverse‐transcribed into cDNA libraries using PrimeScript™ RT Master Mix and sequenced with the Illumina Novaseq™ 6000 (Modi *et al*., 2021). The transcriptome data have been submitted to the National Center for Biotechnology Information (NCBI) Sequence Read Archive (SRA) under accession number PRJNA1130097.

Using HISAT2 version 2.0.4 (Kim *et al*., 2015), the transcriptomic sequencing data were aligned to the genome of *Brassica rapa* (V1.5). Following the alignment analysis, StringTie (Pertea *et al*., 2015) was employed to assemble the aligned reads. The assembled transcripts were then compared with the existing genomic annotation to identify annotated transcriptional regions as well as previously unannotated regions, thereby uncovering novel transcripts and genes in the species. The discovered genes were subjected to sequence alignment against the NR (Deng *et al*., 2006), Gene ontology (GO) (Ashburner *et al*., 2000) and KEGG (Kanehisa *et al*., 2008) databases using the BLAST software (Altschul *et al*., 1997). KOBAS 2.0 (Mao *et al*., 2005) was used to obtain the KEGG orthology results for the genes. Fragments per kilobase of transcript per million fragments mapped (FPKM) (Florea *et al*., 2013) was employed as a metric to measure the expression level of transcripts or genes. DESeq2 (Love *et al*., 2014) was utilized for differential expression analysis between sample groups. A fold change of ≥2 and false discovery rate (FDR) <0.01 were used as the screening criteria. Fold change represents the ratio of expression levels between two samples (groups). The FDR is obtained by adjusting the significance *P*‐values. Pearson's Correlation Coefficient (Sedgwick, 2012) was used to analyse the correlation between the transcriptomic sequencing results and the qRT‐PCR results.

### Sequence and expression pattern analysis

To investigate the structural features and physicochemical properties of *BrCNGC12* and *BrCNGC16*, we designed specific primers to clone their open reading frame sequences (Table S1). A phylogenetic tree including BrCNGC12, BrCNGC16 and CNGC proteins from other species was generated using the neighbour‐joining method in MEGA 6.0 (Tamura *et al*., 2011). The transmembrane topology of BrCNGC12 was predicted using the TMHMM Server v.2.0 (https://services.healthtech.dtu.dk/service.php?TMHMM‐2.0). The conserved domains of the BrCNGC12 and BrCNGC16 proteins were examined using SMART (http://smart.embl‐heidelberg.de/) (Letunic *et al*., 2012). Using the ‘HK‐8’ Chinese cabbage variety as the experimental material, the relative expression levels of *BrCNGC12* and *BrCNGC16* in various tissues at the seedling, heading and early podding stages were quantified by qRT‐PCR. Furthermore, expression differences of *BrCNGC12* and *BrCNGC16* under low‐calcium (LCa) and medium‐calcium (MCa) conditions were assessed.

### Construction of subcellular localization vectors and their transient transformation in tobacco

The open reading frame (excluding the stop codon) of the cloned *BrCNGC16* was inserted into the *Nde* I and *BamH* I restriction sites of the pRI101 vector using homologous recombination, and the resulting construct was named pRI101‐*BrCNGC16*. Additionally, the constructed recombinant vector and the empty vector pRI101 were transformed into *Agrobacterium tumefaciens* GV3101 using the freeze–thaw method (Jyothishwaran *et al*., 2007), and transient expression was performed in tobacco according to the method of Wu *et al*. (2010). After 48 h of dark culture, cellular fluorescence was observed using laser confocal microscopy. The empty pRI101 vector served as a control in the experiment.

### Construction of overexpression vectors and genetic transformation in *Arabidopsis thaliana*

Using pEASY‐Blunt‐*BrCNGC16* as a template, a cDNA fragment containing *Kpn* I and *Spe* I restriction sites was amplified by designing specific primers (Table S1). The amplified fragment was ligated into the pTCK303 vector digested with *Kpn* I and *Spe* I using T4 DNA ligase, and then transformed into *Escherichia coli*. positive clones were selected, cultured and the bacterial lysate was subjected to PCR and sequencing. The recombinant vector with the correct sequence was named pTCK303‐*BrCNGC16*.

Transgenic *A. thaliana* plants were obtained using the *Agrobacterium*‐mediated freeze–thaw method (Jyothishwaran *et al*., 2007) and the floral dip method (Clough and Bent, 1998). Subsequently, the plants were self‐pollinated for multiple generations, with seeds collected from individual plants, ultimately resulting in the T3 generation of homozygous lines. To identify *BrCNGC16* transgenic *A. thaliana* plants, this study first extracted DNA from the leaves and used PCR to detect the hygromycin resistance gene. Next, GUS histochemical staining was employed to assess the staining pattern in the plants. Finally, qRT‐PCR was used to determine the relative expression level of the *BrCNGC16* gene to ensure its overexpression.

### Construction of *BrCNGC12* /*16* silencing vectors and genetic transformation

A specific 40 bp fragment of the *BrCNGC12* gene (TTGATTCTCCTAAGTTCTGCTTCACTTTCGACAAGAAGCT) was identified through sequence analysis. This fragment, along with its reverse complementary sequence, was combined to create an 80 bp DNA fragment, which was then phosphorylated. The vector pTY was digested with *SnaB* I restriction enzyme and dephosphorylated. The phosphorylated DNA fragment was ligated into the dephosphorylated pTY vector using T4 ligase, transformed into *DH5α* cells, screened and sequenced to obtain positive single clones. The positive single clones were cultured for plasmid extraction for future use. The pTY‐*Brcngc12* silenced Chinese cabbage plants were obtained using the gene gun method and the friction method (Yu *et al*., 2018). The specific fragment for *BrCNGC16* was (ATGATTTGGTTTGTGATCCCAAATGCGGGAGAATTCAGAT), and its silencing method was the same as that for *BrCNGC12*. To identify the *Brcngc12*/*16*‐silenced Chinese cabbage plants, the disease symptoms were observed 20 days after virus inoculation to ensure successful infection. Additionally, qRT‐PCR was used to detect the relative expression levels of the *BrCNGC12*/*16* genes to confirm partial silencing of *BrCNGC12*/*16*.

### Genetic transformation of *BrCNGC16* in Chinese cabbage

To obtain overexpression Chinese cabbage plants of *BrCNGC16*, an improved *Agrobacterium*‐mediated genetic transformation method based on Li *et al*. (2021) was used, with the cotyledons and petioles of Chinese cabbage ‘HK‐8’ as explants to acquire *BrCNGC16* overexpression Chinese cabbage plants.

To identify the *BrCNGC16* Chinese cabbage plants, DNA from the transgenic leaves was used as a template, and specific primers were designed to detect the hygromycin resistance gene and the target gene (amplifying parts of the target gene and the vector). Additionally, GUS histochemical staining was used to detect the staining pattern in the plants. Finally, qRT‐PCR technology was employed to measure the relative expression level of the *BrCNGC16* gene.

### Ca^2+^ deficiency treatment and index determination of genetically modified materials

Using *BrCNGC12*/*16* transgenic plants and control plants as materials, treatments with medium calcium (MCa, 5 mmol/L) and low calcium (LCa, 0.01 mmol/L) were established. When the symptoms of tipburn just appeared in some of the plants, the roots and leaves from each treatment were collected to measure the following indices.

#### Statistics of tipburn incidence rate

Overall incidence rate: The percentage of plants exhibiting tipburn symptoms in a line, relative to the total number of plants in that line (Kuronuma *et al*., 2018).

#### Determination of Ca^2+^ content and chlorophyll a content

To determine the Ca^2+^ content, digestion was carried out according to the method of Yuan *et al*. (2021). The digested solution was then diluted to 100 mL with water, and the Ca^2+^ concentration was measured using an inductively coupled plasma atomic emission spectrometer (ICP‐AES). The chlorophyll a content was determined according to the previous research method by Chen *et al*. (2024).

#### The anatomical structure of Chinese cabbage leaves

To analyse the cellular structure of plant leaves, we will perform hand‐cut sections of the inner leaves after 21 days of LCa treatment, including areas with tipburn symptoms and the critical boundary region between affected and unaffected tissue. The specific sectioning method follows the previously established protocol by Yuan *et al*. (2021). The cells will be observed and photographed under an Olympus BX51 microscope.

#### Other physiological indicators

The activities of peroxidase (POD), superoxide dismutase (SOD), catalase (CAT), relative electrical conductivity and nitroblue tetrazolium (NBT) staining were all measured according to previously established methods (Beauchamp and Fridovich, 1971; Ranieri *et al*., 2000; Shen *et al*., 1996; Zhou *et al*., 2007).

### Quantitative real‐time PCR

Gene‐specific primers were designed using Primer 5.0 software (Ren *et al*., 2004) (Table S2). About 1 μg RNA was used as a template to synthesize cDNA using the Primer Script^RT^ Reagent Kit (Takara, Dalian, China). The cDNA was diluted to a certain fold and then used for qRT‐PCR reactions with the ABI Step One Plus system. The reaction system and conditions were based on a previous study in Chinese cabbage (Yuan *et al*., 2021). The Chinese cabbage *Bra028615* gene or the *A. thaliana AT3G13920* gene was used as an internal control gene (Table S2), and the $2^{-\Delta \Delta C_{T}}$ method (Livak and Schmittgen, 2001) was applied to normalize the expression levels of the target genes.

### Statistical analysis methods

GraphPad Prism 8.0 and Microsoft Excel 2016 were used for data analysis and figure generation. All experimental data are presented as the mean standard deviation from at least three independent biological replicates. One‐way ANOVA was used to analyse the data, and the Tukey–Kramer multivariate range test was performed using GraphPad Prism 8.0 (GraphPad Software Inc., San Diego, CA, USA) or DPS software. A *P* value of less than 0.05 was considered statistically significant.

## Conflicts of interest

The authors declare no conflicts of interest.

## Author contributions

Jingping Yuan and Changwei Shen conceived and designed the experiments, Ruixiang Chen and Yunduan Qin performed the experiments. Shuai Li, Bo Sun and Chunyang Feng performed the qRT‐PCR. Jingping Yuan and Xinlei Guo wrote the manuscript. All authors read and approved the final manuscript.

## Supporting information


**Figure S1** Functional annotation and pathway analysis of DEGs.
**Figure S2** Analysis of FPKM values and qRT‐PCR results for 10 genes in DEGs.
**Figure S3** Prediction of the conserved domains of BrCNGC12.
**Figure S4** Prediction of the secondary structure and transmembrane domains of BrCNGC12.
**Figure S5** Positive identification of *BrCNGC16* transgenic *A. thaliana*.

**Table S1** Primer list used for cloning and vector construction of all genes involved in this experiment.
**Table S2** List of quantitative real‐time PCR primers for all genes.
**Table S3** Summary of the RNA‐Seq data collected from the leaves of ‘HK‐8’ under MCa and LCa treatments.
**Table S4** Detailed information on 61 genes related to Ca^2+^ absorption and transport.
**Table S5** Summary of the sources of all BrCNGC16 homologous proteins.

## Data Availability

All data are available within this manuscript and its supporting information. Sequence data from this article can be found in the National Center for Biotechnology Information (NCBI) Sequence Read Archive (SRA) under accession number PRJNA1130097.
