# Supplementary material for: BrCNGC12 and BrCNGC16 mediate Ca2+ absorption and transport to enhance resistance to tipburn in Chinese cabbage
Source: Plant Biotechnol J. 2025 May 3;23(7):2871–87. doi: 10.1111/pbi.70113 (PMC12205870; doi:10.1111/pbi.70113)
Supplement: Supplementary file 1 — Figure S1 Functional annotation and pathway analysis of DEGs. Figure S2 Analysis of FPKM values and qRT‐PCR results for 10 genes in DEGs. Figure S3 Prediction of the conserved domains of BrCNGC12. Figure S4 Prediction of the secondary structure and transmembrane domains of BrCNGC12. Figure S5 Positive identification of BrCNGC16 transgenic A. thaliana. [file PBI-23-2871-s001.zip › Legends.docx]

**Fig. S1** Functional annotation and pathway analysis of DEGs. (a) Gene ontology (GO) analysis of DEGs. (b) KEGG pathway analysis of DEGs. (c) Pathway enrichment of DEGs.

**Fig. S2** Analysis of FPKM values and qRT-PCR results for 10 genes in DEGs. (a) FPKM values and qRT-PCR analysis for *Bra000889*. (b) FPKM values and qRT-PCR analysis for *Bra001676*. (c) FPKM values and qRT-PCR analysis for *Bra005131*. (d) FPKM values and qRT-PCR analysis for *Bra015009*. (e) FPKM values and qRT-PCR analysis for *Bra018089*. (f) FPKM values and qRT-PCR analysis for *Bra020564*. (g) FPKM values and qRT-PCR analysis for *Bra027981*. (h) FPKM values and qRT-PCR analysis for *Bra031515*. (i) FPKM values and qRT-PCR analysis for *Bra033745*.

**Fig. S3** Prediction of the conserved domains of BrCNGC12.

**Fig. S4** Prediction of the secondary structure and transmembrane domains of BrCNGC12. (a) Secondary structure of BrCNGC12. (b) Prediction of the transmembrane domains of BrCNGC12.

**Fig. S5** Positive identification of *BrCNGC16* transgenic *A. thaliana*. (a) Hygromycin resistance gene detection in the leaves of transgenic and WT *A. thaliana* plants. Marker (M) is 2000 bp. From bottom to top, the sizes are 100, 250, 500, 750, 1000, and 2000 bp; P represents the plasmid (positive control). (b) GUS histochemical staining of the leaves from transgenic and WT *A. thaliana* plants. WT, wild-type plant; OE1, OE2 represent transgenic plants.
