# Supplementary material for: BrCNGC12 and BrCNGC16 mediate Ca2+ absorption and transport to enhance resistance to tipburn in Chinese cabbage
Source: Plant Biotechnol J. 2025 May 3;23(7):2871–87. doi: 10.1111/pbi.70113 (PMC12205870; doi:10.1111/pbi.70113)
Supplement: Supplementary file 1 — Figure S1 Functional annotation and pathway analysis of DEGs. Figure S2 Analysis of FPKM values and qRT‐PCR results for 10 genes in DEGs. Figure S3 Prediction of the conserved domains of BrCNGC12. Figure S4 Prediction of the secondary structure and transmembrane domains of BrCNGC12. Figure S5 Positive identification of BrCNGC16 transgenic A. thaliana. [file PBI-23-2871-s001.zip › pbi70113-sup-0003-FigureS3.pdf]

MNTLRGNWRKTILFVCVVALGIDPLYLFVPVVDSPKFCFTFDKKLATGV

SVLRTIIDVFYVVHIIWNFMRYSNGELNLHGKPKREKYYFISYTIVDILSVL

PMPQVLVLTLMRRSDSLVSREILKWIVLSQYIPRIVRIYPLYKEVTKASGT

Ion_trans domain

VAETKWIGAAFNLFLYMLHSHVFGAFWYVSSVEKINKCWRLACPHLP

GCSLKFQYCGREGGNNIPYGLNTSCPLIEPDQITNSSVFNFGMYIDALKS

GIVEVNPDDFPRKFFYCFWWGLRNISALGQNLRTSNSVGDIVFALIICV

SGLLLFAVLIGNIQKYLQSNTTRIDENEEKKRDTEIWMSSKSLPENLKN

MIRSHEEEKWQKTRGIEEKKFLDDLPDHIRLHIERNSLNNVSSLYPC
